# Glucose Deficiency Elevates Acid-Sensing Ion Channel 2a Expression and Increases Seizure Susceptibility in Temporal Lobe Epilepsy

**DOI:** 10.1038/s41598-017-05038-0

**Published:** 2017-07-19

**Authors:** Haitao Zhang, Guodong Gao, Yu Zhang, Yang Sun, Huanfa Li, Shan Dong, Wei Ma, Bei Liu, Weiwen Wang, Hao Wu, Hua Zhang

**Affiliations:** Department of Neurosurgery, Tangdu Hospital, The Fourth Military Medical University, Xi’an, Shaanxi 710038 People’s Republic of China

## Abstract

Brain hypometabolism is a common epilepsy-related finding in both patients and animal models. Fluorodeoxyglucose positron emission tomography studies have shown that recurrent seizures lead to reduced glucose metabolism in certain brain regions, but no studies have definitively determined whether this induces epileptogenesis. There is evidence that acid-sensing ion channel 2a (ASIC2a) affects epilepsy susceptibility. Transcription factor CP2 (TFCP2) regulates ASIC2a expression. We report that suppressed TFCP2 expression and elevated ASIC2a expression were associated with glucose hypometabolism in the hippocampi of humans with epilepsy and of rat epilepsy model brains. In cultured PC12 cells, we determined that glucose deficiency led to TFCP2 downregulating ASIC2a. Moreover, electrophysiological recordings from cultured rat hippocampal slices showed that ASIC2a overexpression resulted in more action potentials in CA1 pyramidal neurons and increased seizure susceptibility. Our findings suggest that hippocampal glucose hypometabolism elevates ASIC2a expression by suppressing TFCP2 expression, which further enhances the intrinsic excitability of CA1 pyramidal neurons and increases seizure susceptibility in patients with temporal lobe epilepsy.

## Introduction

Temporal lobe epilepsy (TLE) is the most common form of drug-resistant epilepsy and is characterised by recurrent and unprovoked seizures^1^. It is diagnosed from medical history, blood tests, and brain structural and functional imaging techniques including electroencephalography (EEG), computed tomography (CT), positron emission tomography (PET), and magnetic resonance imaging (MRI). The cellular uptake of fluorine-18-tagged fluorodeoxyglucose (^18^F-FDG), the most common radiopharmaceutical in PET imaging, is a marker for tissue metabolic activity. Seizure foci frequently appear hypometabolic (i.e., having reduced glucose utilisation) in interictal PET scans^2, 3^. Many studies have reported that recurrent seizures cause considerable hypoxia^4^, regional ischaemia^5^, and mitochondrial dysfunction^6, 7^, all of which reduce glucose utilisation. However, Pumain *et al*.^8^ suggested that low glucose utilisation might instead be related to dysfunctional endogenous γ-aminobutyric acid-A (GABA_A_) receptor phosphorylation that results in greater lability of neuronal inhibition. Whatever its source, it remains unclear whether decreased glucose utilisation contributes to epileptogenesis.

An imbalance of neuronal excitation and inhibition is thought to cause epilepsy. Acid-sensing ion channels (ASICs), which are proton-gated cation channels, are strongly linked to abnormal neuronal excitability^9, 10^. These Na^+^-conducting channels (ASIC1a also conducts Ca^2+^) can depolarise the membrane and thereby influence neuronal function and excitability^11, 12^. ASIC1a, ASIC2a, and ASIC2b are the most commonly-expressed subunits of the trimeric ASIC receptor in the central nervous system. The ASIC antagonist amiloride suppresses generalised seizures induced by both maximal electrical stimulation and pentylenetetrazole. It also delays the onset of the first seizure episode and the occurrence of status epilepticus (SE) following pilocarpine administration^13^. ASIC inhibition diminishes epileptic discharges in hippocampal slices in a low-Mg^2+^ epilepsy model and markedly reduces kainate-induced discharges in the hippocampus *in vivo* ^14^. Additionally, the ASIC1a rs844347-A allele frequency is higher in patients with epilepsy than in healthy controls, and haplotype analysis has revealed that this allele is significantly associated with TLE^15^. In our previous study, we showed that ASIC expression patterns in the piriform cortex are altered after seizures, which further suggests that ASIC2a expression increases susceptibility to epilepsy^16^.

Transcription factor CP2 (TFCP2, also known as late SV40 factor) is highly conserved, has a very old lineage, and regulates the expression in haematopoietic cells^17, 18^, of immune-related genes^19^ and many other viral genes^20^. TFCP2 binds the ASIC2a gene’s core promoter segment such that mutations at the binding site result in a 95% loss of basal promoter activity in a luciferase reporter assay^21^. Strong upregulation of TFCP2 has been reported in cell lines and samples from patients with hepatocellular carcinoma^22^. Additionally, hepatocellular carcinoma is associated with glucose hypermetabolism^23^. Thus, the data suggest that low glucose utilisation may alter TFCP2 expression and, consequently, ASIC2a expression.

Here, we examined changes in TFCP2 and ASIC2a expression in relation to low glucose utilisation in both tissues and cells, as well as the relationship between TFCP2 and ASIC2a. We also explored how modulating ASIC2a expression affects the intrinsic excitability of CA1 pyramidal neurons and how hippocampal transfection of ASIC2a-expressing adeno-associated viruses (AAVs) affected seizure susceptibility in rats.

## Results

### Abnormal hippocampal TFCP2 and ASIC2a expression and glucose hypometabolism in patients with TLE

**Figure 1:**
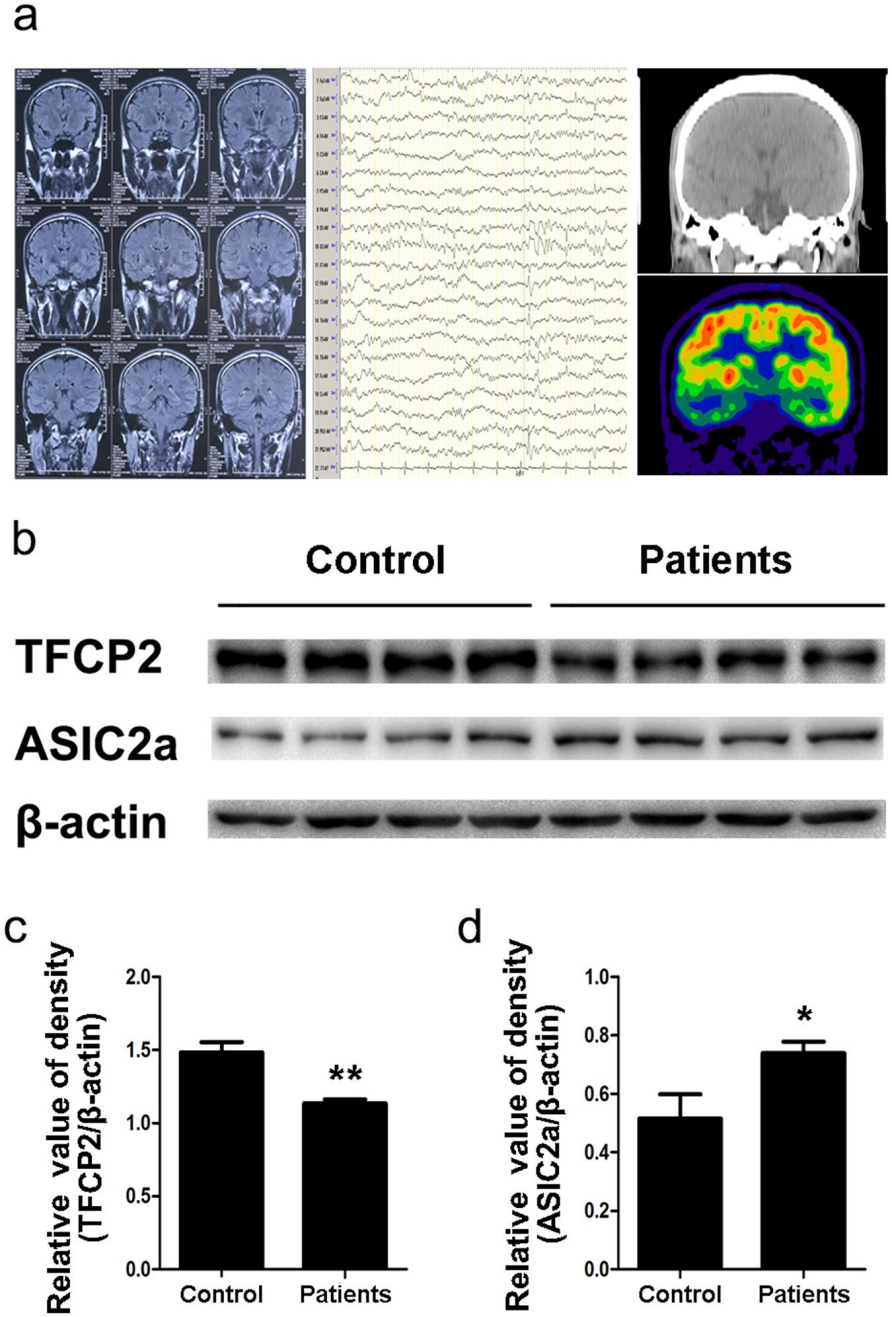
Altered hippocampal TFCP2 and ASIC2a expression with glucose hypometabolism in patients with TLE. (**a**) Patient 4’s pre-surgical assessment results: magnetic resonance imaging (left) was negative, electroencephalography (middle) showed spike waves in the temporal lobe, and fluorodeoxyglucose positron emission tomography (right) revealed hypometabolic lesions in the right hippocampus. (**b**) Representative western blot assays of hippocampal TFCP2 and ASIC2a expression in patients with TLE (n = 13) and control patients (n = 10). β-actin was used as a loading control. (**c**,**d**) Normalised densitometry bar graphs of TFCP2 and ASIC2a for the control subjects and patients with TLE. The experiments were repeated at least 3 times. Data are presented as means ± standard errors and were analysed using unpaired t-tests. *P < 0.05, **P < 0.01 compared to controls. Uncropped western blot images are shown in Supplementary Fig. 1. Abbreviations, ASIC2a: acid-sensing ion channel 2a; TFCP2: transcription factor CP2; TLE: temporal lobe epilepsy.

Brain samples from 13 patients with intractable epilepsy and 10 control subjects were collected in this study. Figure 1a shows the inclusion criteria for patients with TLE: negative MRI results, abnormal EEG in the temporal lobe and hippocampus, and PET evidence of unilateral hypometabolic lesions in the hippocampus. Tissue samples including zones of hippocampal hypometabolism in patients with TLE were analysed via western blotting (Fig. 1b). The patients’ TFCP2 intensity ratios were significantly lower than those of the control subjects (control subjects: 1.484 ± 0.070; patients: 1.135 ± 0.027; P < 0.01; Fig. 1c). Additionally, the patients had significantly higher ASIC2a expression levels than the control subjects did (control subjects: 0.515 ± 0.083; patients: 0.739 ± 0.039; P < 0.05; Fig. 1d).

### Abnormal hippocampal TFCP2 and ASIC2a expression and glucose hypometabolism in epileptic rats

**Figure 2:**
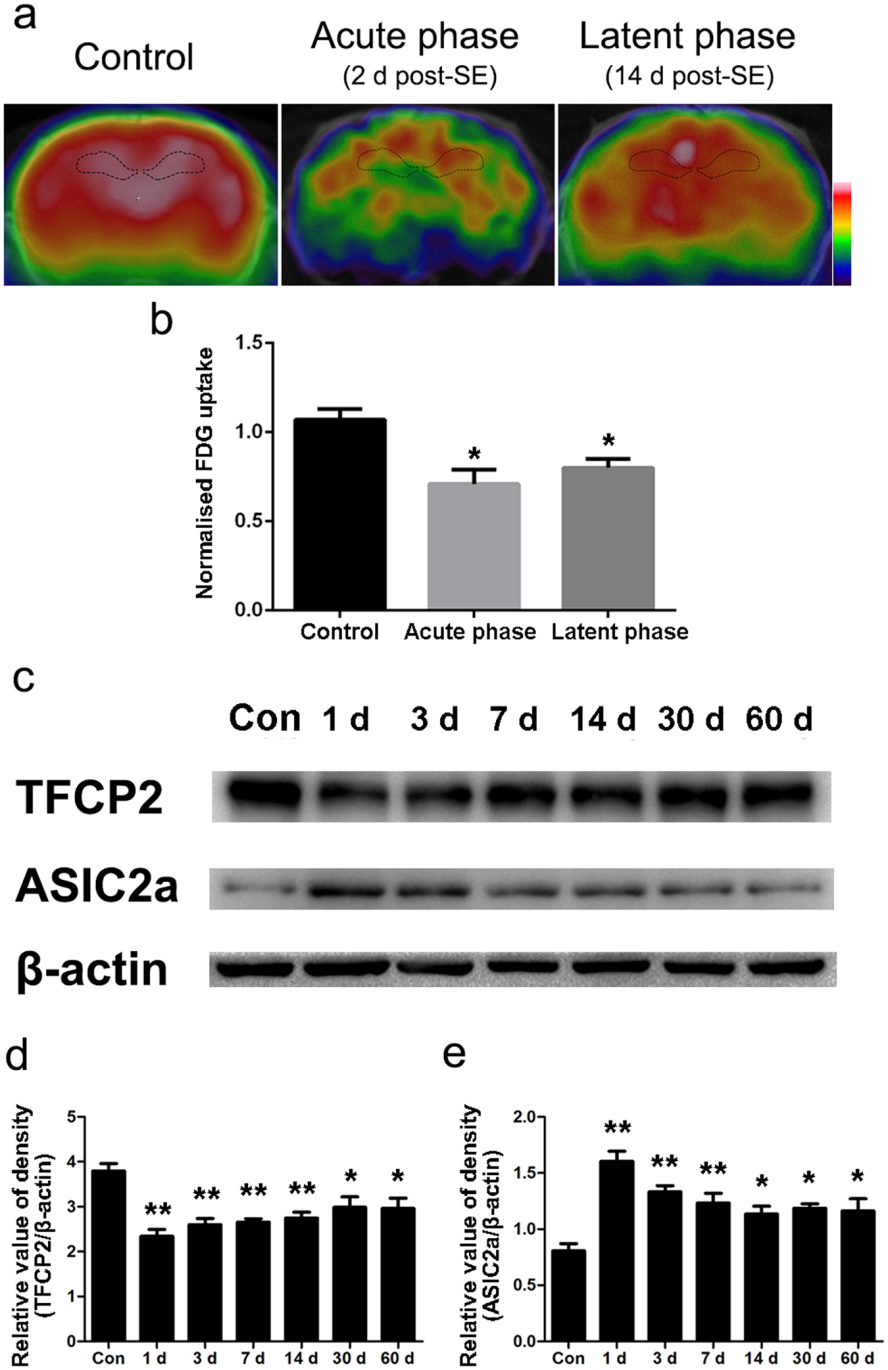
Altered hippocampal TFCP2 and ASIC2a expression with glucose hypometabolism in pilocarpine-treated rats. (**a**) Representative coronal view microscopic positron emission tomography images in the different phases of epileptogenesis. (**b**) Hippocampal glucose uptake in the different phases after pilocarpine injection. (**c**) Representative western blot assays of hippocampal TFCP2 and ASIC2a expression at different post-seizure time points. (**d**) Bar graph showing decreased hippocampal TFCP2 expression in pilocarpine-treated rats. (**e**) Bar graph showing increased hippocampal ASIC2a expression in pilocarpine-treated rats. The experiments were repeated at least 3 times with at least 3 rats in each group. Data are presented as means ± standard errors and were analysed using 1-way ANOVA and Dunnett’s multiple comparisons test. *P < 0.05 compared with controls, **P < 0.01 compared with controls. Uncropped western blot images are shown in Supplementary Fig. 1. Abbreviations, ASIC2a: acid-sensing ion channel 2a; TFCP2: transcription factor CP2; SE: status epilepticus; Con: control.

We measured TFCP2 and ASIC2a expression in pilocarpine-treated rats, a well-established animal model of TLE. The rats went through an acute phase (4 hours to 3 days post-SE), a seizure-free latent phase (7–21 days post-SE), and a chronic phase with spontaneous recurrent seizures (28–60 days post-SE)^24–26^. FDG-microPET scans showed hippocampal glucose hypometabolism during the acute and latent phases (Fig. 2a). In the acute phase, the pilocarpine-treated rats had significantly lower normalised FDG uptake values than the control rats (33% reduction, P < 0.05). Hippocampal glucose uptake partially recovered in the latent phase but was still 25% lower than the pre-treatment baseline (P < 0.05) (Fig. 2b). Western blotting revealed that TFCP2 expression was significantly decreased 1, 3, 7, and 14 days post-seizure (P < 0.01 for all time points); partially recovered during the chronic phase; but then remained significantly lower than that of the controls (P < 0.05) (Fig. 2c,d). Additionally, ASIC2a expression was significantly increased 1, 3, and 7 days post-seizure (P < 0.01 for all time points). It decreased slightly during the chronic phase but remained significantly higher than that of the controls (P < 0.05) (Fig. 2c,e).

### Glucose deficiency in PC12 cells influenced TFCP2 and ASIC2a expression

**Figure 3:**
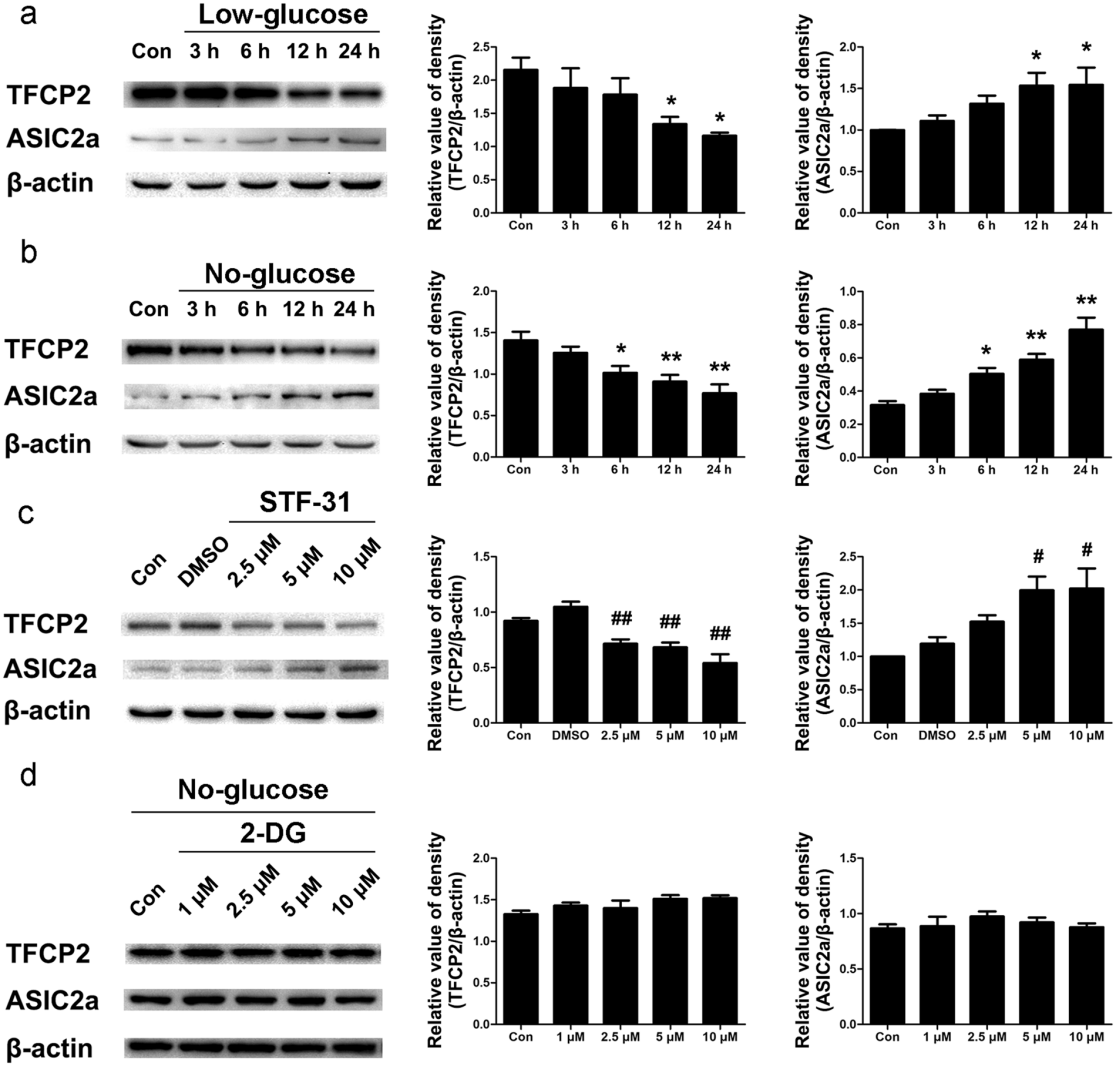
Glucose deficiency influenced TFCP2 and ASIC2a expression in PC12 cells. (**a**) Representative immunoblot and densitometric analyses showing that cells grown in low-glucose media had significantly decreased TFCP2 expression and significantly increased ASIC2a expression relative to those grown in high-glucose media after 12 and 24 h of growth. (**b**) Representative immunoblot and densitometric analyses showing that cells grown in no-glucose media had significantly decreased TFCP2 expression and significantly increased ASIC2a expression relative to those grown in high-glucose media after 6, 12, and 24 h of growth. (**c**) Representative immunoblot and densitometric analyses showing that STF-31-treated PC12 exhibited significant downregulation of TFCP2 and significant upregulation of ASIC2a relative to DMSO-treated control cells. (**d**) Representative immunoblot and densitometric analyses showing TFCP2 and ASIC2a expression in 2-deoxy-D-glucose-treated PC12 cells cultured in no-glucose media. The experiments were repeated at least 3 times. Data are presented as means ± standard errors and were analysed using 1-way ANOVA and Dunnett’s multiple comparisons test. *P <  0.05, **P <  0.01 compared to controls; ^#^P < 0.05, ^##^P < 0.01 compared to the DMSO group. Uncropped western blot images are shown in Supplementary Fig. 1. Abbreviations, ASIC2a: acid-sensing ion channel 2a; TFCP2: transcription factor CP2; DMSO: dimethyl sulfoxide; 2-DG: 2-deoxy-D-glucose; Con: control.

Cellular FDG uptake is a marker for tissue glucose utilisation and overall glucose metabolism. Glucose metabolism is related to glucose supply, glucose transportation, glycolysis, and aerobic oxidation^27^. To determine whether TFCP2 and ASIC2a expression were related to glucose deficiency, we simulated different glucose utilisation conditions in PC12 cells. At both the 12 and 24 h collection points, TFCP2 expression was significantly lower in cells grown in low-glucose media than in those grown in high-glucose media (P < 0.05), but ASIC2a expression was significantly higher in the cells grown in low-glucose media (P < 0.05) (Fig. 3a). At the 6 h collection point, the cells grown in no-glucose media also exhibited significantly lower TFCP2 expression and significantly higher ASIC2a expression than those grown in high-glucose media (P < 0.05) (Fig. 3b).

The glucose transporter I (GLUT1) inhibitor STF-31 reduces cellular glucose uptake^28^. STF-31-treated cells exhibited lower TFCP2 expression than vehicle (dimethyl sulfoxide, DMSO)-treated cells, and cells treated with 5 or 10 µM STF-31 exhibited higher ASIC2a expression (P < 0.05) (Fig. 3c). This suggests that both TFCP2 and ASIC2a expression are related to cytoplasmic glucose concentrations. 2-deoxy-D-glucose (2-DG) is a glucose analogue that cannot be further metabolised by phosphoglucose isomerase (GPI). Interestingly, adding 2-DG to the no-glucose media did not change TFCP2 or ASIC2a expression levels within 24 h (P > 0.05) (Fig. 3d), but adding 10 µM 2-DG to the high-glucose media suppressed TFCP2 expression and increased ASIC2a expression within 24 h (P < 0.05) (see Supplementary Fig. 2). Glucose and 2-DG were simultaneously transported into cells, thereby reducing cytoplasmic glucose concentrations. This suggests that changes in TFCP2 and ASIC2a expression may be related to glycolysis itself or intermediate products of glycolysis after GPI enzyme activity.

### TFCP2 downregulated ASIC2a expression

**Figure 4:**
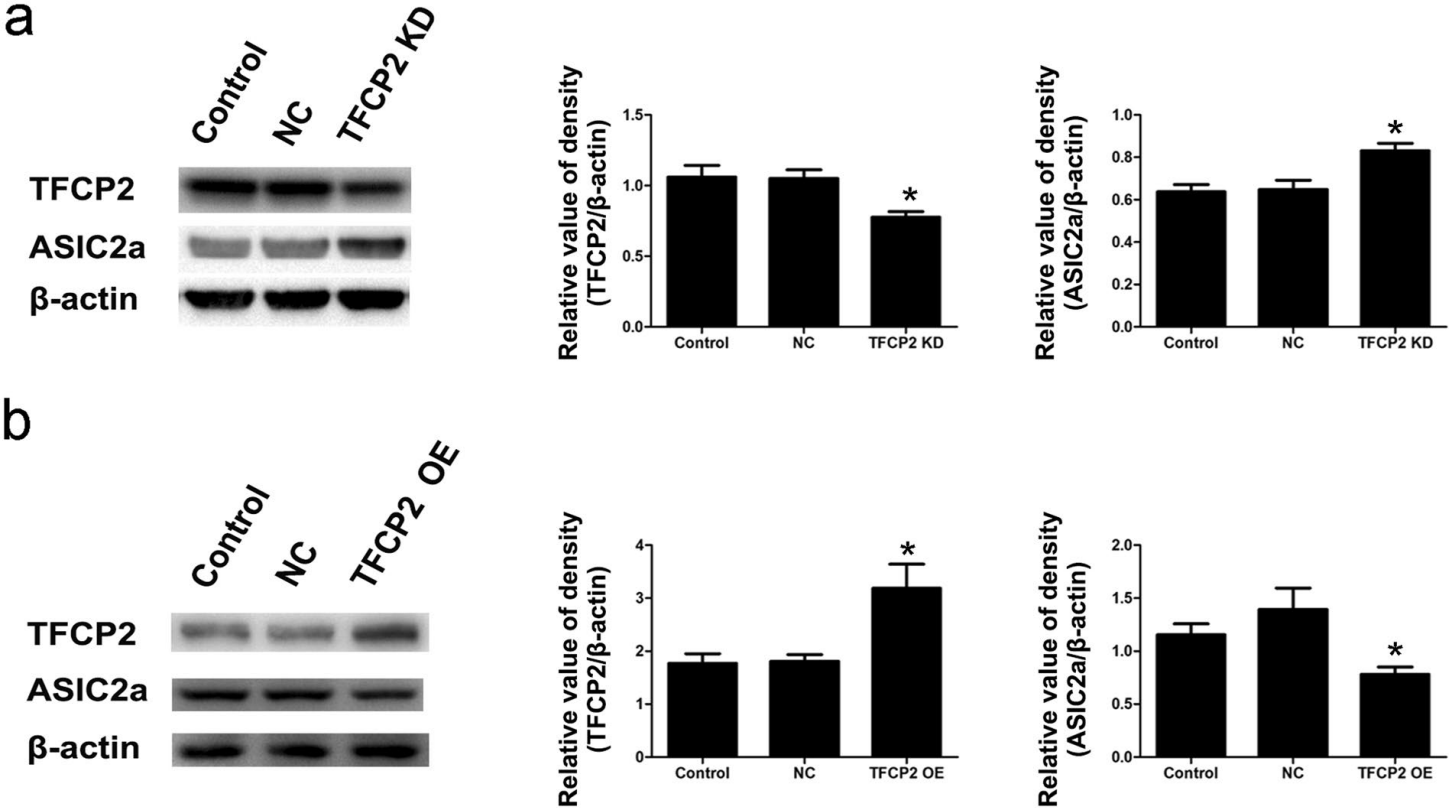
TFCP2 inversely regulates ASIC2a expression. (**a**) Representative immunoblot and densitometric analyses showing that TFCP2 siRNA-transfected PC12 cells had 27% lower TFCP2 expression than negative siRNA-treated cells and consequently elevated ASIC2a expression. **(b**) Representative immunoblot and densitometric analyses showing that TFCP2 OE plasmid-transfected PC12 cells had 1.76 ± 0.45-fold greater TFCP2 expression than negative control cells and consequently suppressed ASIC2a expression. The experiments were repeated at least 3 times. Data are presented as means ± standard errors and were analysed using unpaired t-tests. *P < 0.05 compared to negative controls. Uncropped western blot images are shown in Supplementary Fig. 1. Abbreviations, ASIC2a: acid-sensing ion channel 2a; TFCP2: transcription factor CP2; siRNA: short interfering RNA; OE: overexpression; KD: knockdown; NC: negative control.

TFCP2 and ASIC2a expression levels were inversely related in the previous experiments, so we hypothesised that TFCP2 downregulates ASIC2a expression. To test this hypothesis, we transfected PC12 cells with TFCP2 short interfering RNA (siRNA) and TFCP2 overexpressing plasmids (Fig. 4a,b). TFCP2 levels in cells transfected with TFCP2-knockdown siRNA exhibited a significant 27% reduction relative to those transfected with a negative siRNA control (P < 0.05). TFCP2 knockdown significantly increased ASIC2a expression (P < 0.05) (Fig. 4a). ASIC2a levels in cells transfected with TFCP2 overexpression plasmids were significantly lower than those in cells transfected with negative plasmids control (P < 0.05) (Fig. 4b).

### TFCP2 and ASIC2a cellular localisation in glucose deficient cells and the CA1 region of epileptic rats

**Figure 5:**
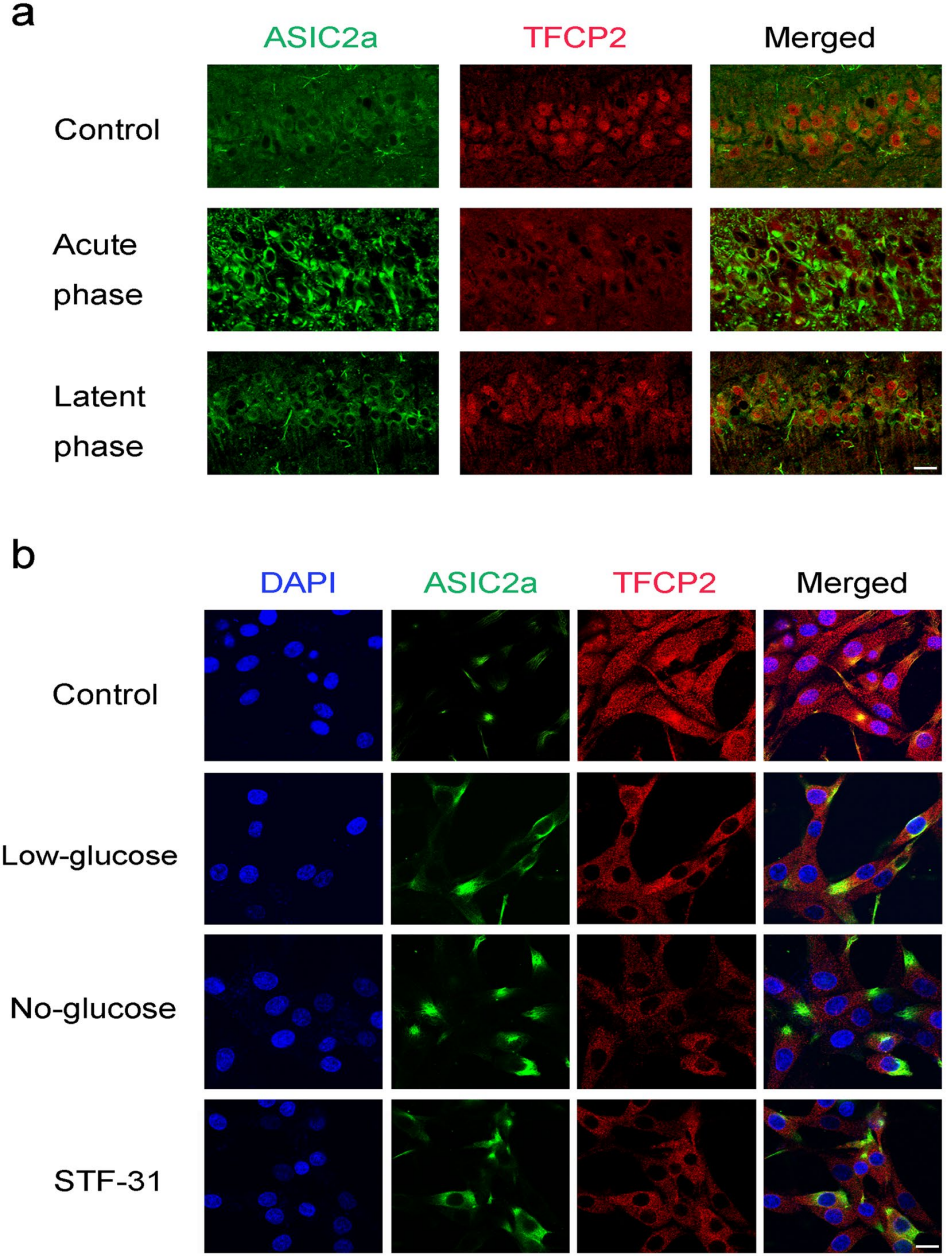
Cellular localisation of TFCP2 and ASIC2a in the epileptic rats’ CA1 regions and glucose-deficient cells. **(a**) Double immunofluorescence labelling for TFCP2 and ASIC2a in the epileptic rats’ CA1 regions during acute and latent post-seizure phases. (**b**) Cellular localisation of TFCP2 and ASIC2a expression in PC12 cells grown for 24 h in high-glucose media (control), low-glucose media, no-glucose media, and STF-31 (10 µM)-loaded media. Proteins were probed with anti-ASIC2a (green) and anti-TFCP2 (red) antibodies. Nuclei were counterstained with DAPI (blue). Scale bars = 20 μm. Abbreviations, ASIC2a: acid-sensing ion channel 2a; TFCP2: transcription factor CP2; DAPI: 4′,6-diamidino-2-phenylindole.

TFCP2 and ASIC2a were immunofluorescently labelled in rat CA1 region pyramidal neurons and PC12 cells. In rats and cultured cells, TFCP2 was detected in the cytoplasm and nucleus, while ASIC2a was mainly expressed in the cytoplasm and plasma membrane (Fig. 5a,b). Interestingly, nuclear TFCP2 expression was greatly reduced in the acute phase of epilepsy (Fig. 5a). Moreover, the PC12 cells exhibited decreased nuclear TFCP2 expression after 24 h of growth in low-glucose media, no-glucose media, or 10 µM STF-31 (Fig. 5b).

### Changes in ASIC2a expression affected the intrinsic excitability of CA1 pyramidal neurons

**Figure 6:**
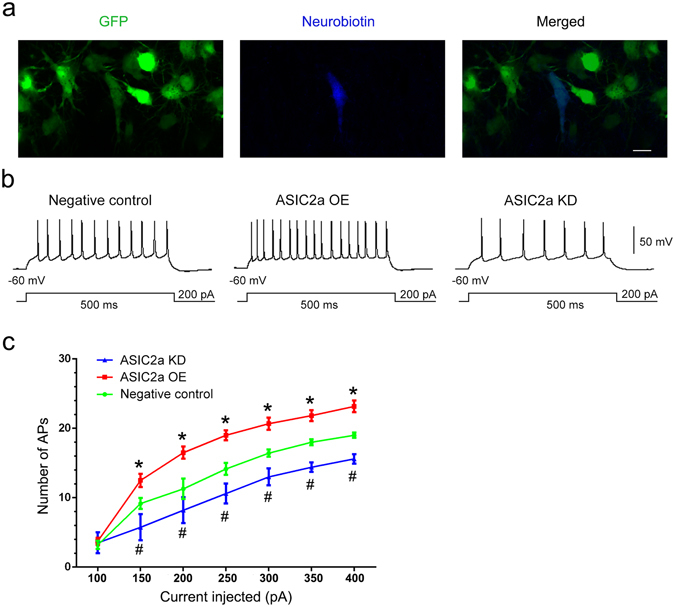
Changes in ASIC2a expression affected the intrinsic excitability of CA1 pyramidal neurons. (**a**) Confocal images of CA1 pyramidal neurons expressing GFP (green) and labelled with neurobiotin (blue). Scale bar = 10 μm. (**b**) Representative traces of action potential firing in response to 200 pA current injections in CA1 pyramidal neurons of the negative control, ASIC2a overexpression, and ASIC2a knockdown groups, respectively. (**c**) Number of action potentials in CA1 pyramidal neurons from the various groups at different current injection steps. Data are presented as means ± standard errors and were analysed using 1- or 2-way ANOVA and Dunnett's multiple comparisons test. *P < 0.05, ASIC2a OE group compared with negative control group; ^#^P < 0.05, ASIC2a KD group compared with negative control group. Abbreviations, ASIC2a: acid-sensing ion channel 2a; GFP: green fluorescent protein; OE: overexpression; KD: knockdown.

ASICs have been shown to regulate neuronal excitability^10, 11^. Hyperexcitation of hippocampal neurons is considered an epileptogenic factor in TLE^29^. To determine whether changes in ASIC2a expression alter hippocampal neuron excitability, we investigated the intrinsic excitability of CA1 region pyramidal neurons in cultured brain slices from rats with altered ASIC2a expression. We used lentiviruses to specifically overexpress or knockdown neuronal ASIC2a and to express green fluorescent protein (GFP) in successfully transfected cells. Neurons were also neurobiotin-labelled during patch-clamp recordings. We analysed data from GFP-expressing, neurobiotin-labelled neurons (Fig. 6a). Action potentials were stimulated with depolarizing current injections. The ASIC2a-overexpressing neurons fired significantly more action potentials than the negative controls at all current injections above 150 pA (P < 0.05). Similarly, ASIC2a knockdown neurons fired significantly fewer action potentials than the negative controls at all current injections above 150 pA (P < 0.05) (Fig. 6b,c). However, we found no significant between-group differences in the input resistance of CA1 pyramidal neurons (see Supplementary Fig. 3). The proton-gated currents recorded in CA1 pyramidal neurons transfected with different lentiviruses are shown in Supplementary Fig. 4. ASIC2a-overexpressing neurons shorten the desensitization rate (τ_d_) of pH 5-induced currents, and ASIC2a knockdown neurons prolonged the desensitization of proton-gated currents.

### Hippocampal ASIC2a overexpression increased seizure susceptibility

**Figure 7:**
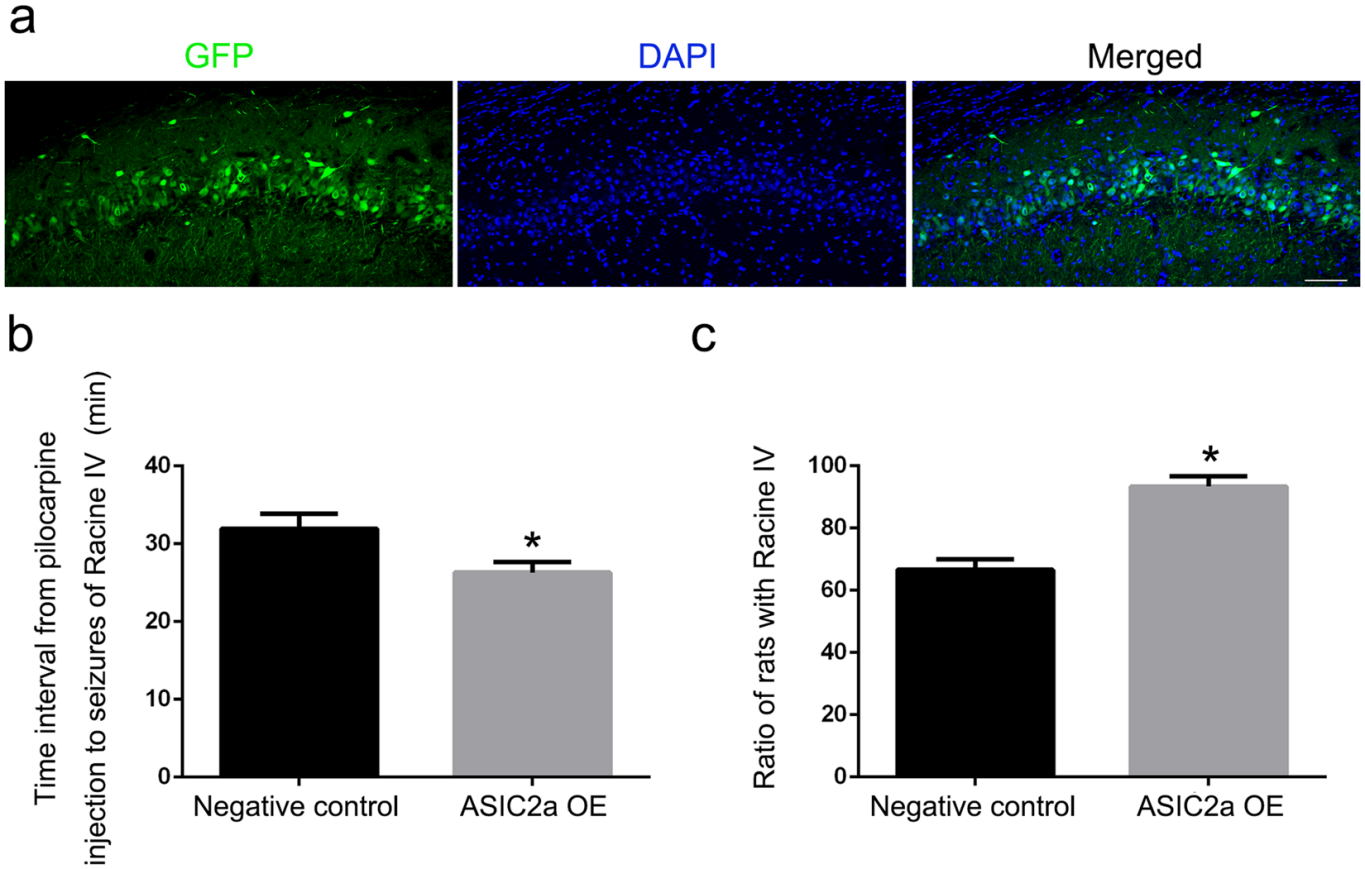
Hippocampal ASIC2a overexpression increased seizure susceptibility. (**a**) Representative images showing GFP immunoreactivity in the hippocampal CA1 region following adeno-associated virus (AAV) vector infusion. Scale bar = 100 μm. (**b**) The time interval from pilocarpine injection to Racine IV seizures was significantly shorter in the ASIC2a overexpression group (26.3 ± 1.3 min) than in the negative control AAV group (31.9 ± 1.9) (n = 30 rats/group). (**c**) The proportion of rats with Racine IV seizures after pilocarpine treatment was significantly higher in the ASIC2a overexpression group (93.3 ± 3.33%) than in the negative control AAV group (66.7 ± 3.33%). Data are presented as means ± standard errors and were analysed using unpaired t-tests or Chi-square tests. *P < 0.05 compared with negative control group. Abbreviations, ASIC2a: acid-sensing ion channel 2a; GFP: green fluorescent protein; DAPI: 4′, 6-diamidino-2-phenylindole; OE: overexpression.

To determine the impact of ASIC2a overexpression on seizure susceptibility *in vivo*, we examined the seizure behaviour of AAV-transfected and negative control rats following pilocarpine administration. We first confirmed AAV vector-induced hippocampal gene expression using immunofluorescence. Confocal images showed GFP expression in virtually all principal hippocampal neurons, as shown for an AAV-injected rat’s CA1 region pyramidal cells in Fig. 7a. In pilocarpine administration tests, ASIC2a overexpression significantly accelerated the onset of the first episode reaching Racine stage IV (P < 0.05) (Fig. 7b) and significantly increased the occurrence rate of SE episodes reaching Racine stage IV (P < 0.05) (Fig. 7c).

## Discussion

Glucose hypometabolism in patients with epilepsy is thought to result from recurrent seizures inducing hypoxia, regional ischaemia, and mitochondrial dysfunction^4–6^, but some studies suggest that it may be related to deficient GABA_A_ receptor phosphorylation and consequently greater lability of GABAergic inhibition^8^. Our data showed reduced hippocampal glucose utilisation in epileptic rats during the acute and latent phases, consistent with previous studies^30, 31^. This suggests that hippocampal hypometabolism occurs before spontaneous epileptic seizures and may therefore induce epileptogenesis.

It is well established that metabolic disturbances influence cellular gene expression patterns by activating multiple intracellular pathways and transcriptional factors^32^. Our data showed that hippocampal TFCP2 expression was suppressed in patients with epilepsy exhibiting glucose hypometabolism, in epileptic rats, and in glucose-deficient PC12 cells. Interestingly, 2-DG did not change TFCP2 expression in PC12 cells grown in no-glucose media. This suggests that TFCP2 may be downregulated in response to a reduction of available glucose in the cytoplasm, or certain intermediate products of glycolysis. Glucose deficiency may thus regulate ASIC2a expression via TFCP2.

TFCP2 is usually a transcriptional activator, both in cell-free extracts and in cultured mammalian cells^20^, but it can also be a transcriptional repressor. For example, TFCP2 represses the HIV long terminal repeat^33^ and downregulates the IL-2 promoter^34^. We found that TFCP2 and ASIC2a expression patterns were inversely related, which suggests that TFCP2 downregulates ASIC2a expression. Xia *et al*.^21^ reported that a mutation in ASIC2a’s TFCP2 binding site reduced a luciferase reporter gene’s basal expression by 95%, but they did not reveal how changes in TFCP2 expression affected intracellular ASIC2a expression. We investigated this question and found that inhibiting TFCP2 expression increased ASIC2a expression in PC12 cells. Reduced TFCP2 expression may thus reduce ASIC2a degradation or indirectly promote the potent ASIC2a transcriptional factors in PC12 cells and epileptic tissues.

ASICs are involved in epileptic seizures and influence epileptogenic susceptibility^9, 10, 13–15^. Our previous study^16^ suggested that ASIC2a overexpression increases susceptibility to stimulated seizures, but in the present study’s pilocarpine administration tests, ASIC2a overexpression accelerated the onset of the first seizure and elevated the SE occurrence rate. Moreover, our patch-clamp recordings showed that ASIC2a overexpression increased the intrinsic excitability of CA1 region pyramidal neurons and that ASIC2a expression knockdown decreased neuronal excitability. Hyperexcitability in the hippocampal CA1 region could therefore trigger epileptogenesis and/or the spreading of epileptic seizures. Although we did not find the clear and precise mechanism by which ASIC2a overexpression increases neuronal excitability, the possible mechanism will be evaluated later.

Due to the relatively deeper position of the patched neurons, it is difficult to record acid-induced currents in acute brain slices. Organotypic brain slice culture was adopted in present study. With the extension of culture time, dead cells and tissue debris disappeared from the surface of the slice, leaving mainly live and healthy cells. Spots of completely “cleaned” and smooth cell somata could be found on the surface of the slice, allowing easy access for patch clamping^35^. Our data of the proton-gated currents in neurons transfected by lentivirus showed that ASIC2a overexpression shortened the time of desensitization of pH 5-induced currents, and ASIC2a knockdown prolonged the time of desensitization process. It suggested that ASIC2a made little contribution to current amplitude, but influenced the desensitization of the currents. Our observation is consistent with previous literatures^36–38^.

Indeed, previous studies showed that seizure termination in acidic environments was related to the activation of ASIC1a^39^ and ASIC3^40^ on interneurons. We therefore believe that ASIC function may be dependent on seizure stage. Before seizure onset, increased ASIC2a expression could increase neuronal excitability and sensitivity to stimulation. As large quantities of lactic acid and glutamic acid are released following seizures, extracellular protons activate ASIC1a and ASIC3 on interneurons, causing GABA release and seizure termination. Further studies are required to explore whether post-seizure brain acidification can activate ASIC2a, leading to seizure termination.

This study has some limitations. Firstly, ethical concerns prevented us from acquiring hippocampal tissue from healthy subjects, so for controls, we used relatively normal brain tissues from patients with head trauma. Secondly, it is difficult to reduce regional glucose supply in the rat hippocampus, so we could not directly observe the relationship between brain glucose hypometabolism and epileptogenesis.

In conclusion, our findings suggest that hippocampal hypometabolism may directly elevate ASIC2a expression by suppressing TFCP2 expression, which subsequently enhances the intrinsic excitability of CA1 pyramidal neurons and ultimately increases susceptibility to TLE. Brain glucose hypometabolism may be another epileptogenic mechanism, consistent with the ‘‘seizures beget seizures’’ hypothesis.

## Methods

### Ethics statement

This study’s human experimental protocols were approved by the Fourth Military Medical University’s Ethics Committee on Human Research. All procedures conformed to the principles of the Declaration of Helsinki. All participants received a complete description of the study and gave written informed consent. The animal experimental procedures were approved by the university’s Committee on Animal Use for Research and Education. Animal experiments were conducted in accordance with the US National Institutes of Health Guide for the Care and Use of Laboratory Animals (NIH Publications No. 80–23, revised 1996).

### Subject recruitment

Epilepsy diagnoses were made according to the criteria established by the International League Against Epilepsy. All patients were diagnosed with TLE based on having recurrent epilepsy and being refractory to the maximal doses of at least 3 anti-epileptic drugs (AEDs). During pre-surgical assessment, the patients were evaluated by multiple methods, including a detailed medical history, a neurological examination, MRI, 18F-FDG-PET, scalp video-EEG monitoring, and intraoperative electrocorticography. MRI results were normal in all included patients, but their FDG-PET results indicated hippocampal hypometabolism lesions. The epileptiform discharge zones and hypometabolic hippocampi were resected for treatment in the university’s Tangdu Hospital Department of Neurosurgery. The postoperative seizure outcome for each patient was classified according to Engel’s classification (class I: seizure-free; class II: fewer than 3 seizures annually; class III: at least an 80% reduction in seizures; and class IV: no significant improvement). The patients’ clinical characteristics are listed in Supplementary Table 1.

Control brain samples were obtained from patients who underwent therapeutic surgical resection due to head trauma. The patients or their legal next of kin gave written consent for the tissues being used for research. None had a history of AED exposure or any known neurological diseases including epilepsy. The control subjects’ clinical characteristics are listed in Supplementary Table 2.

### Animals and treatments

All efforts were made to minimise the number of animals and their suffering. Male Sprague–Dawley rats (6- to 7-week old, 200–240 g body weight) were provided by the university’s Experimental Animal Centre. They were housed under standard environmental conditions (temperature: 22 ± 1 °C, humidity: 50–60%, and 12-h light/dark cycle) with ad libitum access to food and water. Lithium chloride (125 mg/kg, Sigma-Aldrich, St. Louis, MO) was intraperitoneally injected 18–20 h prior to an intraperitoneal injection of pilocarpine (40 mg/kg, Sigma-Aldrich). Methscopolamine (1 mg/kg in 0.9% saline, Sigma-Aldrich) was intraperitoneally administered 30 min before pilocarpine administration to minimise the pilocarpine’s peripheral effects. After 1 h of SE, diazepam (10 mg/kg) was intraperitoneally injected to terminate seizures, and, if necessary, 2 to 3 additional injections (5 mg/kg) were administered to improve survival rates. To prevent dehydration, all experimental animals received intraperitoneal saline (0.9%, 1 mL) injections immediately following a seizure and twice on the day following a seizure. Control rats were treated identically except for receiving saline (0.9%) instead of pilocarpine. All animals received special care until they were sacrificed. The evoked behavioural seizures were scored using Racine’s scale for quantitative seizure intensity descriptions (stage I: mouth and facial movement; stage II: head nodding; stage III: forelimb clonus; stage IV: rearing with forelimb clonus; and stage V: rearing and falling with forelimb clonus). Only animals that reached stage IV were used for subsequent experiments. At scheduled time points after pilocarpine injection, some of the animals were used for 18F-FDG-microPET imaging, some were sacrificed by decapitation and had their brains rapidly extracted for western blotting, and the rest were perfused and fixed with paraformaldehyde for immunofluorescence analysis.

### 18F-FDG-PET imaging in patients

As mentioned earlier, all TLE patients underwent FDG-PET scanning during their non-invasive pre-surgical evaluations. We acquired images during the interictal period using a General Electric PET/CT scanner (GE Healthcare, Chicago, IL). We first acquired head CT scans for attenuation correction (120 keV, 200 mA, 1.0-s rotation time, 512 × 512 matrix, 0.8 pitch, and 3-mm slice thickness) and then acquired brain PET scans at least 30 min after intravenous injection of 18F-FDG (0.1 mCi/kg).

### 18F-FDG-microPET imaging in rats

Images were acquired from 3 groups, each containing 3 rats with similar weights: (1) saline-treated rats (control), (2) pilocarpine-treated rats 2 days after SE (acute phase), and (3) pilocarpine-treated rats 14 days after SE (latent phase). Under brief isoflurane anaesthesia, the rats received caudal vein injections of 18F-FDG (500 µCi) and were then placed in a quiet location before being returned to individual cages. The rats were allowed to move freely for 60 min following the injection. Then, 10-min static scans and helical CT images were acquired under isoflurane anaesthesia using a microPET/CT scanner (Mediso, Budapest, Hungary). PET and CT images were automatically fused using image fusion software (Mediso).

### PET data analysis

Glucose uptake was calculated in regions of interest drawn in the bilateral hippocampus, including the dorsal and ventral hippocampus, for 3 slices. The FDG-PET image analysis was conducted as previously described^41^. The calculated cerebellum activity was used as the normalisation reference. Each normalised FDG-PET volume was reformatted by linear extrapolation to have the same voxel size as the CT volumes. The reformatted FDG-PET images were then manually co-registered to corresponding CT images. For patients, a standard uptake value difference of more than 10% was considered an indication of glucose hypometabolism, and we compared hippocampal metabolism in the 2 hemispheres. For rats, we compared the 3 groups previously described.

### Antibodies and reagents

The proteins were probed with anti-TFCP2 (Cat. No. 610818, BD Biosciences, Franklin Lakes, NJ), anti-ASIC2a (Cat. No. ab169768 and ab77384, Abcam, Cambridge, UK), and anti-β-actin (Cat. No. 04–1116, Millipore, Billerica, MA) antibodies and 4′,6-diamidino-2-phenylindole (DAPI, Cat. No. 2871890–3, Millipore). The secondary antibodies included goat anti-rabbitimmunoglobulin G (IgG) conjugated to horseradish peroxidise (HRP, Cat. No. ab6721, Abcam), goat anti-mouse IgG conjugated to HRP (Cat. No. ab97023, Abcam), goat anti-rabbit IgG H&L (Alexa Fluor® 488, Cat. No. ab150077, Abcam), and goat anti-mouse IgG H&L (Alexa Fluor®647, Cat. No. ab150115, Abcam).

All chemicals were purchased from Sigma-Aldrich. STF-31, bicuculline, CGP55845, and 6,7-dinitroqui-noxaline-2,3-dione (DNQX) were dissolved in DMSO. 2-DG and D-2-amino-5-phosphonopentanoate (D-AP5) were dissolved in deionised water. The drugs for cultured cells were dissolved to the desired final concentrations in the media. The drugs for the electrophysiological experiments were dissolved to the desired final concentrations in the artificial cerebrospinal fluid (aCSF).

Negative control siRNA, TFCP2-specific siRNA, negative control plasmids, and TFCP2-overexpressing plasmids were bought from GenePharma (Shanghai, China). The TFCP2 siRNA’s sense strand was 5′-GACCUGGAGACAGAAUUCUTT-3′, and its antisense strand was 5′-AGAAUUCUGUCUCCAGGUCTT-3′.We bought GFP-only, ASIC2a knockdown, and ASIC2a overexpression lentiviruses from Genechem (Shanghai, China). The GFP-only lentiviruses were the negative controls. The ASIC2a target sequence was 5′-AACCATCAGCCACACTGTGAA-3′. The ASIC2a-affecting lentiviruses contained a GFP reporter gene. AAV vectors expressing ASIC2a with or without GFP were also bought from Genechem.

### Tissue preparation

Human tissues were immediately frozen in liquid nitrogen and stored at −80 °C for western blotting. Animal tissues were immediately divided into 1 portion that was immediately frozen in liquid nitrogen and then stored at −80 °C for western blotting and a second portion that was fixed in 4% phosphate-buffered formalin for 48 h. This portion was frozen and cut into 15-μm sections with a cryostat microtome (Leica CM1860, Germany) for double immunofluorescence labelling and stored at −20 °C.

### Cell culture

PC12 cells were plated on 60-mm dishes and incubated at 37 °C in a moist 5% CO_2_, 95% air atmosphere. The media was changed every 48 h until the cells were ready for use. To decrease glucose supply, the high-glucose Dulbecco’s Modified Eagle’s Medium (DMEM) (glucose: 4500 mg/L, GE Healthcare) was replaced with low-glucose DMEM (glucose: 1000 mg/L, GE Healthcare) or no-glucose DMEM (glucose: 0 mg/L, GE Healthcare) when the cells became 80% confluent. Cells were collected after 3, 6, 12, and 24 h of growth, and cells grown in high-glucose media were used as controls. PC12 cells were collected 24 h after treatment with the GLUT1 inhibitor STF-31 (2.5, 5, or 10 µM). 2-DG (1, 2.5, 5, or 10 µM) was added to the high-glucose and no-glucose media for 24 h.

### Western blotting

Total protein extracts from tissues or cell were separated by 10% SDS-PAGE and electroblotted onto a polyvinylidene difluoride (PVDF) membrane (Millipore) at 200 mA for 180 min. Nonspecific binding sites were blocked by immersing the PVDF membrane in 1% bovine serum albumin blocking solution (Tris-buffered saline/Tween 20 solution). Proteins were probed overnight at 4 °C with anti-ASIC2a, anti-TFCP2, and anti-β-actin antibodies and then probed at room temperature for 2 h with secondary antibodies (goat anti-mouse IgG conjugated to HRP and goat anti-rabbit IgG conjugated to HRP). The images were collected with the ChemiDoc XRS+ system (Bio-Rad, Hercules, CA).

### Immunofluorescence labelling

The hippocampal sections from rats were fixed in 4% paraformaldehyde for 10 min, permeabilised with 0.4% Triton X-100 for 15 min, and incubated in 5% bovine serum albumin (MP Biomedicals, CA) for 30 min at 37 °C. The sections were then incubated overnight at 4 °C with a mixture of rabbit polyclonal anti-ASIC2a antibody (1:100, Abcam) and mouse monoclonal anti-TFCP2 antibody (1:100, BD Biosciences). They were then incubated in a darkroom for 90 min at 37 °C with goat anti-rabbit IgG H&L (Alexa Fluor® 488, Cat. No. ab150077, Abcam) and goat anti-mouse IgG H&L (Alexa Fluor®647, Cat. No. ab150115, Abcam) antibodies and mounted in 1:1 glycerol/phosphate-buffered saline (PBS). The sections from AAV-injected rats were obtained 3 weeks after AAV infusion. Nuclei were stained with DAPI (300 nM) for 15 min.

PC12 cells were fixed in 4% paraformaldehyde for 20 min, permeabilised with 0.4% Triton X-100 for 15 min, and incubated with 5% goat serum for 30 min at 37 °C. The cells were strained as before. Nuclei were stained with DAPI (300 nM) for 15 min and then rinsed in PBS. Confocal images were acquired with a Nikon C2^+^ confocal microscope system (Nikon, Japan).

### siRNA and plasmid transfections

The PC12 cells were transfected with siRNA or plasmids using the Lipofectamine™ 2000 transfection agent (Invitrogen, Carlsbad, CA). In brief, 2.0 × 10^5^ PC12 cells were seeded onto 60-mm diameter plates with media containing 10% foetal bovine serum (Cat. No. 10100147, Thermo-Fisher, Waltham, MA). The cells were transfected at 60–70% confluence. The transfection reagents and either siRNA or plasmids were added to the serum-free media for 4 h. The cells were incubated for another 48 h (for plasmid transfection) or 24 h (for siRNA transfection) in serum-containing regular media. The cells were then collected for western blotting.

### Organotypic slice cultures and lentivirus infection

Hippocampal slices were cultured as previously described^35^. Briefly, P3–4 Sprague–Dawley rat pups of both genders were fully anaesthetised and decapitated. Their brains were rapidly sectioned in cold sterile slicing media with a tissue slicer (model HA752, Campden Instruments, Lafayette, IN). Serial coronal slices (300 μm) of the hippocampal CA1 regions were prepared. The slices were separated under a dissecting microscope and halved at the midline. For each experiment, at least 18 slices from3 animals were used. Six hippocampal slices were transferred onto a 0.4-μm Millicell-CM membrane insert (Millipore) in a 6-well plate. The slices were maintained in culture media (1 mL, 50% MEM media, 25% horse serum, and 25% Hanks’ balanced salt solution; Gibco-BRL system, Thermo-Fisher) supplemented with D-glucose (5 mg/mL) and L-glutamine (2 mm) at 37 °C. The media was changed thrice weekly.

We injected virus solution (0.1–0.2 μL) into the extracellular space of the cultured slices’ pyramidal cell layers 3 days after the start of cultivation. Electrophysiological recordings were performed after the slices had been cultured for 8 days.

### Electrophysiological recordings

Patch-clamp recordings were conducted as previously described^42^. CA1 region pyramidal neurons were identified under infrared differential interference contrast microscopy (Olympus). Whole-cell patch clamp recordings were obtained using a MultiClamp 700B amplifier (Axon Instruments, Union City, CA). The aCSF contained NaCl (124 mm), KCl (3 mm), NaHCO_3_ (24 mm), CaCl_2_ (2 mm), NaH_2_PO_4_ (1.25 mm), MgSO_4_ (1 mm), and D-glucose (10 mm) and was equilibrated with carbogen gas. CA1 neurons were patched with 3–6 MΩ micropipettes pulled with a P-97 puller (Sutter Instruments, Novato, CA). The internal pipette solution contained K-gluconate (145 mm), NaCl (5 mm), 4-(2-hydroxyethyl)-1-piperazineethanesulfonic acid (free acid, 10 mm), ethylene glycol-bis(β-aminoethyl ether)-N,N,N′,N′-tetraacetic acid (0.2 mm), Na-guanosine-5′-triphosphate (0.3 mm), and Mg-adenosine triphosphate (4 mm) and had a pH of 7.3 and an osmolarity of 280–290 mOsm. The recordings were filtered at 3 kHz, digitised at 10 kHz, and analysed with pCLAMP10.2 software (Axon Instruments). Series resistance and whole cell capacitance were continually monitored and compensated for throughout the experiment. All potentials were corrected for the junction potential online by adjusting the pipette’s offset using the MultiClamp700B control software. The recording chamber was maintained at 37 °C with a line heater (TC-324B, Warner Instruments, Hamden, CT). We only recorded from neurons with a resting membrane potential (RMP) more negative than −50 mV and a series resistance that varied by less than 20%. GABAergic and glutamatergic transmission were blocked by supplementing the bath solution with bicuculline (10 µM), CGP55845 (1 µM), DNQX (10 µM), and D-AP5 (50 µM). In current-clamp mode, RMP was measured at each recording’s beginning. Current was injected to maintain the membrane voltage at −60 mV. Membrane input resistance (R_in_) was measured as the reciprocal conductance slope of the linearly regressed steady-state voltage responses elicited by a series of low-amplitude (−50 to +30 pA), 500-ms square current steps. Action potentials were stimulated by injecting 500-ms depolarizing current steps from 100 pA to 400 pA at an interval of +50 pA. In recording of proton-gated currents, SF-77B perfusion system (Warner Instruments, USA) was used (see Supplementary methods for details). This system was widely applied to record acid-induced currents^43, 44^. The solution change could be completed in milliseconds.

The pipette also contained 1% neurobiotin tracer (SP-1155, Vector Laboratories, Burlingame, CA), which passively diffused into cells. After recordings, the slices were imaged using a Nikon C2^+^ confocal microscope system (Nikon, Japan) that counted the GFP-expressing, neurobiotin-labelled neurons.

### AAV vector administration and behaviour analysis

The rats were anaesthetised with intraperitoneally injected pentobarbital (70 mg/kg). We bilaterally injected either AAV-GFP or AAV-ASIC2a (2 µL for either) into the dorsal hippocampus (−3.8 mm posterior to bregma, ±2 mm lateral to the midline and −3 mm ventral of the dorsal surface of the skull) using a stereotaxic frame (RWD Life Science, Shenzhen, China). Behavioural tests began 4 weeks after vector infusion when transgene protein expression had peaked and stabilised. The rats’ seizures were observed after pilocarpine injections (40 mg/kg). The time from the injection to reaching Racine stage IV was also recorded.

### Statistical analysis

All data are presented as means ± standard errors (n = number of samples). Statistical analyses were performed in GraphPad Prism 6.0 (GraphPad Software, San Diego, CA). Unpaired t tests were used to compare the patients’ protein expression levels (Fig. 1), while 1-way ANOVA was used to compare the rats’ protein expression levels at different time points (Fig. 2) and the protein expression levels in *in vitro* experiments (Fig. 3). Unpaired t tests were used to analyse the regulatory functions of TFCP2 on ASIC2a (Fig. 4). For the electrophysiology data, 2-way ANOVA was used to analyse the number of induced APs (Fig. 6). Unpaired t-tests or Chi-square tests were used to analyse the seizure susceptibility test data (Fig. 7). We defined statistical significance as P < 0.05 and used Dunnett’s multiple comparison test for all ANOVA comparisons.

## Electronic supplementary material

Supplementary Information
