# Supplementary material for: Glucose Deficiency Elevates Acid-Sensing Ion Channel 2a Expression and Increases Seizure Susceptibility in Temporal Lobe Epilepsy
Source: Sci Rep. 2017 Jul 19;7:5870. doi: 10.1038/s41598-017-05038-0 (PMC5517604; doi:10.1038/s41598-017-05038-0)
Supplement: Supplementary file 1 — Supplementary Information [file 41598_2017_5038_MOESM1_ESM.pdf]

Supplementary Information

Glucose Deficiency Elevates Acid-Sensing Ion Channel 2a Expression and Increases Seizure Susceptibility in Temporal Lobe Epilepsy

Haitao Zhang[§] · Guodong Gao[§] · Yu Zhang[§] · Yue Zhang · Huanfa Li · Shan Dong · Wei Ma · Bei Liu · Chao Wang · Hao Wu [*] · Hua Zhang[*]

Department of Neurosurgery, Tangdu Hospital, The Fourth Military Medical University, Xi'an, Shaanxi 710038, People's Republic of China

[§]Haitao Zhang, Guodong Gao and Yu Zhang contributed equally to this work.

Correspondences and requests for materials should be addressed to:

Professor Hua Zhang
Department of Neurosurgery
Tangdu Hospital, The Fourth Military Medical University
Xin-si Road, Xi'an, Shaanxi 710038, China
Tel: +86-29-84777286
Fax: +86-29-84777435
E-mail: zhanghua@fmmu.edu.cn

Mr Hao Wu
Department of Neurosurgery
Tangdu Hospital, The Fourth Military Medical University
Xin-si Road, Xi'an, Shaanxi 710038, China
Tel: 86-29-84717824
Fax: +86-29-84777435
E-mail: wuhaohb@163.com

SUPPLEMENTARY INFORMATION

Table of contents:

- Supplementary Methods
- Supplementary Table (1-2)
- Supplementary Figures (1-4)

Supplementary Methods

Patch-clamp recordings of proton-activated currents in CA1 pyramidal neurons

Electrophysiological recordings were performed after hippocampal slices had been transfected with lentivirus and in culture for 8 days. Whole-cell patch clamp recordings were obtained using MultiClamp 700B amplifier (Axon Instr., USA). Data were filtered at 3 kHz, digitized at 10 kHz, and analyzed by using pCLAMP10.2 software (Axon Instr., USA). The pipette solution contained (in mM): 120 KCl, 10 NaCl, 2 $MgCl_2$, 5 EGTA, 2 ATP, 10 HEPES. The pH was adjusted with tetramethylammonium hydroxide. Extracellular solutions contained (in mM): 128 NaCl, 5.4 KCl, 5.55 glucose, 10 HEPES, 10 MES, 2 $CaCl_2$, and 1 $MgCl_2$. The pH was adjusted with tetramethylammonium hydroxide, and osmolarity was adjusted with tetramethylammonium chloride. Experiments were carried out at room temperature (20-24°C). The bath solution was supplemented with bicuculline (10 μM), CGP55845 (1 μM), DNQX (10 μM) and D-AP5 (50 μM) to block GABAergic and glutamatergic transmission. Electrodes had a resistance of 3-5 MΩ, and series resistance was compensated by 70% after establishing the whole-cell configuration. Neurons were held at -70 mV, and changes in extracellular pH were induced by shifting a microperfusion system (SF-77B, Warner Instruments) in front of the cell. Amplitude was determined by subtracting the baseline current at pH 7.4 from the peak current amplitude determined in Clampfit (Axon Instruments). Capacitance was measured for each neuron in Clampex (Axon Instruments). The tau of desensitization (τ_d) was calculated by fitting the data to a single exponential. Statistical significance was evaluated using the 1-way ANOVA and Dunnett's multiple comparisons test.

Supplementary Table 1. Clinical characteristics of TLE patients

patient	gender	age at surgery (years)	duration of epilepsy (years)	AEDs before surgery	MRI	PET	surgery type	Neuropathological diagnosis	Engel class
1	M	26	16	CBZ, PHT, VPA	N	LTH	LT+AH	G	I
2	F	22	20	OXC, VPA, LEV	N	LTH	LT+AH	NL, G	I
3	F	52	29	CBZ, VPA, PHT, PB	N	LTH	LT+AH	NL, G	II
4	M	42	12	VPA, CBZ, LTG	N	RTH	RT+AH	NL, G	I
5	M	28	25	PHT, CBZ, PB, CZP	N	LTH	LT+AH	G	II
6	M	21	16	CBZ, VPA, LGT	N	RTH	LT+AH	NL, G	I
7	M	22	9	CBZ, VPA, LGT	N	RTH	LT+AH	G	I
8	M	22	5	VPA, LEV, OXC	N	LTH	LT+AH	G	II
9	M	21	19	CBZ, TPA, VPA	N	RTH	RT+AH	NL, G	I
10	M	20	8	VPA, CBZ, PB, PHT	N	RTH	RT+AH	G	I
11	M	21	4	CBZ, LEV, VPA	N	RTH	RT+AH	NL, G	I
12	M	29	11	PHT, PB, CBZ, VPA	N	RTH	RT+AH	NL, G	I
13	M	24	13	VPA, OXC, LTG	N	LTH	LT+AH	G	II

M, male; F, female; N, normal; VPA, valproate; PB, phenobarbital; LEV levetiracetam; CBZ, carbamazepine; TPM, topiramate; PHT, phenytoin; LTG, lamotrigine; GBP, gabapentin; OXC, oxcarbazepine; CZP, clonazepam; LT+AH, left temporal and amygdalohippocampectomy; LTH, left temporal hypometabolism; RTH, right temporal hypometabolism; LT+AH, left temporal and amygdalohippocampectomy; RT+AH, right temporal and amygdalohippocampectomy; NL, neuronal loss; G, gliosis

Supplementary Table 2. Clinical characteristics of control group

Patient	Gender	Age (years)	Etiology diagnosis	Resection tissue	Adjacent tissue pathology
1	M	35	trauma	TNr	N
2	M	32	trauma	TNl	N
3	M	24	trauma	TNr	N
4	F	22	trauma	TNl	N
5	F	29	trauma	TNr	N
6	M	21	trauma	TNl	N
7	M	18	trauma	TNr	N
8	M	43	trauma	TNl	N
9	M	27	trauma	TNl	N
10	M	29	trauma	TNl	N

M, male; F, female; TN, temporal neocortex; l, left; r, right; N, normal.

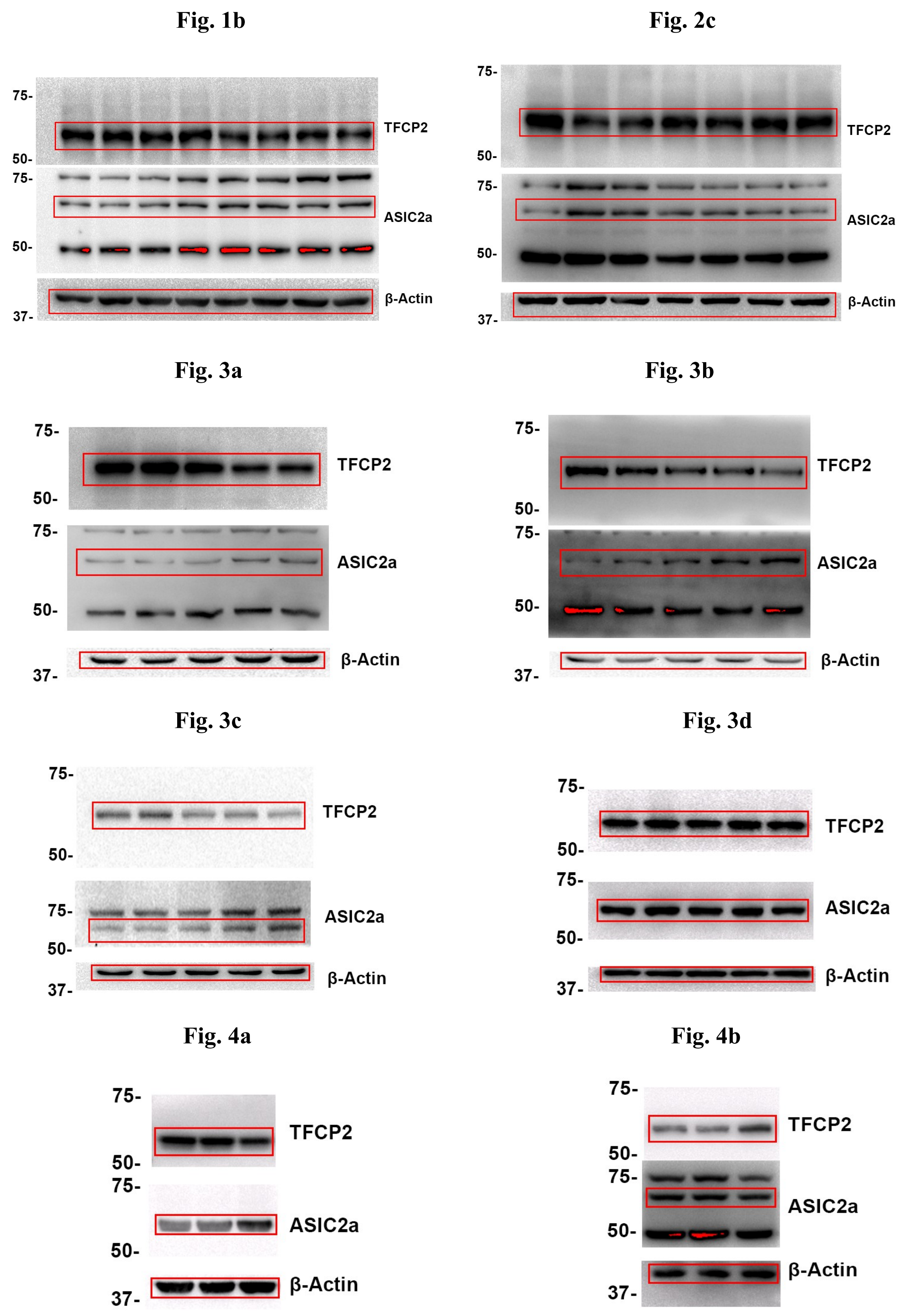

Supplementary Figure 1. Uncropped, full-size images of the original scans of western blots shown in Fig. 1b, 2c, 3a, 3b, 3c, 3d, 4a and 4b.

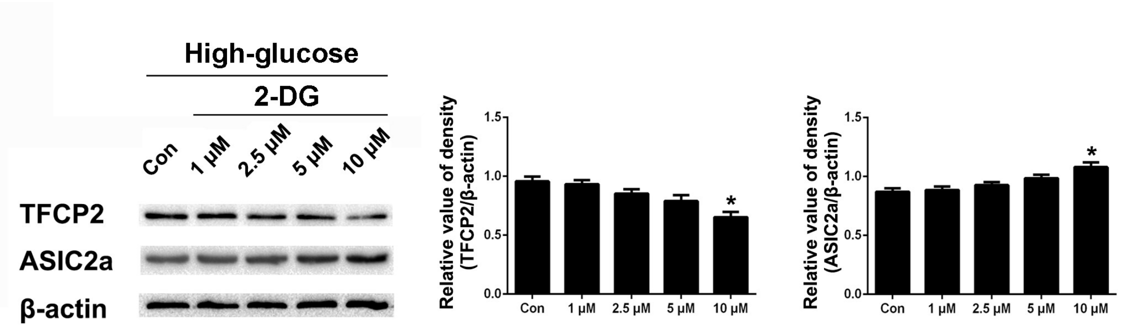

Supplementary Figure 2. TFCP2 and ASIC2a expression in 2-DG-treated PC12 cells cultured with high glucose media. In the high-glucose condition, TFCP2 expression was suppressed, while ASIC2a expression was elevated in PC12 cell treated with 10 μM 2-DG for 24 hours. Data are presented as means ± standard errors and were analysed using 1-way ANOVA and Dunnett's multiple comparisons test. *$P<0.05$, compared to controls. Abbreviations, ASIC2a: acid-sensing ion channel 2a; TFCP2: transcription factor CP2; 2-DG: 2-deoxy-D-glucose; Con: control

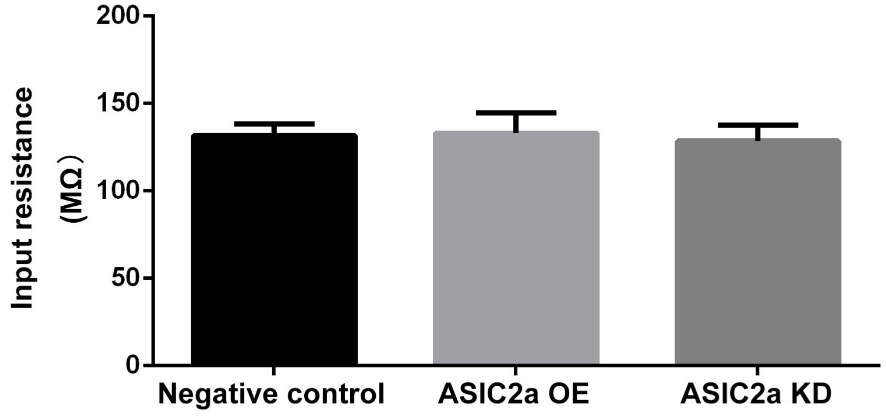

Supplementary Figure 3. Input resistance of CA1 pyramidal neurons transfected by lentivirus. There were no differences in the input resistance of CA1 pyramidal neurons between all groups. Data are presented as means ± standard errors and were analysed using 1-way ANOVA and Dunnett’s multiple comparisons test. Abbreviations, ASIC2a: acid-sensing ion channel 2a; OE: overexpression; KD: knockdown

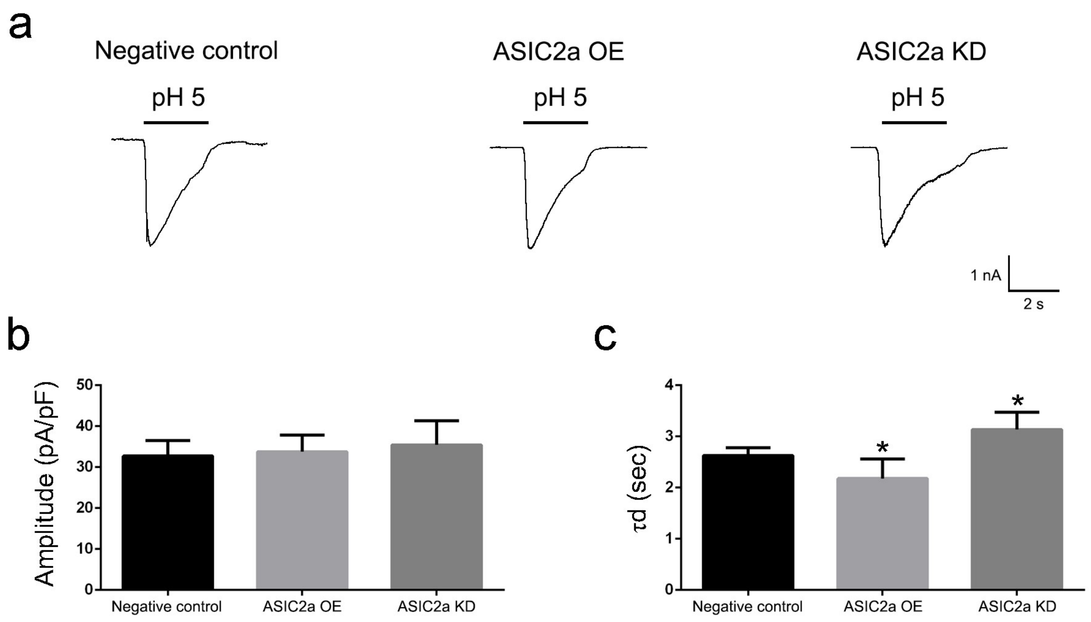

Supplementary Figure 4. Proton-gated currents from CA1 pyramidal neurons with different ASIC2a expression pattern. **(a)** Representative trace of pH 5-induced current in neurons transfected with different lentivirus. **(b)** Average peak amplitude of pH 5-induced current. **(c)** Desensitization rate of pH 5-induced currents. Data are from pyramidal neurons transfected with negative control lentivirus (n=6), ASIC2a overexpression lentivirus (n=7), and ASIC2a knockdown lentivirus (n=6). Data are presented as means ± standard errors and were analysed using 1-way ANOVA and Dunnett's multiple comparisons test, *P<0.05, compared to negative control. Abbreviations, ASIC2a: acid-sensing ion channel 2a; OE: overexpression; KD: knockdown
